# Super-Resolution ^1^H Magnetic Resonance Spectroscopic Imaging Utilizing Deep Learning

**DOI:** 10.3389/fonc.2019.01010

**Published:** 2019-10-09

**Authors:** Zohaib Iqbal, Dan Nguyen, Gilbert Hangel, Stanislav Motyka, Wolfgang Bogner, Steve Jiang

**Affiliations:** ^1^Medical Artificial Intelligence and Automation Laboratory, Department of Radiation Oncology, University of Texas Southwestern Medical Center, Dallas, TX, United States; ^2^Christian Doppler Laboratory for Clinical Molecular MR Imaging, Department of Biomedical Imaging and Image-guided Therapy, High Field MR Center, Medical University of Vienna, Vienna, Austria

**Keywords:** super-resolution, magnetic resonance spectroscopic imaging (SI), deep learning (DL), magnetic resonance spectroscopy (^1^H MRS), artificial intelligence

## Abstract

Magnetic resonance spectroscopic imaging (SI) is a unique imaging technique that provides biochemical information from *in vivo* tissues. The ^1^H spectra acquired from several spatial regions are quantified to yield metabolite concentrations reflective of tissue metabolism. However, since these metabolites are found in tissues at very low concentrations, SI is often acquired with limited spatial resolution. In this work, we test the hypothesis that deep learning is able to upscale low resolution SI, together with the T1-weighted (T1w) image, to reconstruct high resolution SI. We report on a novel densely connected UNet (D-UNet) architecture capable of producing super-resolution spectroscopic images. The inputs for the D-UNet are the T1w image and the low resolution SI image while the output is the high resolution SI. The results of the D-UNet are compared both qualitatively and quantitatively to simulated and *in vivo* high resolution SI. It is found that this deep learning approach can produce high quality spectroscopic images and reconstruct entire ^1^H spectra from low resolution acquisitions, which can greatly advance the current SI workflow.

## 1. Introduction

Magnetic resonance imaging (MRI) continues to be a versatile modality capable of providing anatomical, metabolic, and functional information from various regions of the body *in vivo*. In particular, magnetic resonance spectroscopic imaging (SI) (1) is able to yield important data regarding the metabolism of different tissues, and has been especially useful for studying the metabolism of the human brain (2). Some important biochemicals, or metabolites, in the brain include N-acetyl aspartate (NAA), glutamate (Glu), glutamine (Gln), creatine (Cr), choline (Ch), and myo-Inositol (mI) (3). Each metabolite plays an important role in regulating energy consumption in the brain, and some metabolites also play critical functional roles, including roles as neurotransmitters (4). It is well-known that metabolic changes occur in parallel with anatomical changes for a myriad of pathologies (2), and these metabolic changes may even occur before structural changes are detected. While SI has continued to be an active area of research over the past several decades, there are still major roadblocks into standardizing this technique and including it into clinical protocols.

One of the major disadvantages of SI is the long acquisition duration associated with obtaining spectra from several voxels of interest. This is primarily due to the fact that many of the important metabolites are found in the brain at low concentrations; these metabolites are typically present in the brain at 1–12 mM concentrations (3). Therefore, in order to accurately detect these biochemicals, several signal averages have to be obtained or larger voxel volumes have to be acquired to improve the signal to noise ratio (SNR) for the experiment. As a result, spatial resolution tends to be coarse for many SI sequences. This low resolution, coupled with other technical problems such as partial volume effects, hinders the overall diagnostic capabilities of the SI technique.

There have been many advances in the technological implementation of SI that allow for faster acquisition and better spatial resolution. One of the primary acceleration methods is echo planar spectroscopic imaging (EPSI) (5, 6), which collects spectral data from an entire line of k-space in a single repetition time (TR) utilizing an echo planar readout. This spatio-spectral acquisition approach has also been applied in non-cartesian SI methods, such as spiral acquisitions (7), concentric circular acquisitions (8), and rosette acquisitions (9). In addition, parallel imaging (10–12) can also be used to accelerate the collection of SI data. Sensitivity encoding (SENSE) has been applied in combination with EPSI (13) to facilitate even faster acquisition times. Recently, research has also focused on the application of various sampling schemes that allow for reduced scan time (14–18). Some studies (19, 20) have even demonstrated protocols capable of obtaining spectroscopic images at 64x64 or 128x128 resolution in less than 20 min. Although these advances have improved the field significantly, SI is still understandably seen as a low SNR, low resolution technique.

In order to combat the limits of the experimentally acquired resolution, many post-processing methods have been developed for super-resolution SI (21–27). These methods have mainly focused on model-based reconstruction methods and regularized reconstruction approaches. While many super-resolution methods are independent of the acquisition protocols, there are some techniques, such as the spectroscopic imaging by exploiting spatio-spectral correlation (SPICE) method (18), that show reconstruction benefits by employing inter-dependent sequences. Unfortunately, the majority of super-resolution methods either tend to be very complicated to implement, or generally show poor reconstruction results. Since experimental acquisitions have many technical challenges, there is also a large concern over the true gold standard for these super-resolution techniques. Without a true standard of comparison, which is a large problem in the spectroscopic imaging field, many studies qualitatively and quantitatively compare their methods with less ideal standards such as bicubic interpolation.

Deep learning is an advancing field that has shown extraordinary results for image processing (28–30). Convolutional layers and networks are capable of extracting valuable features from images, and can further process these features into labels or other images for classification, segmentation, and other uses. One network that has been extremely beneficial for the field of automated medical imaging segmentation is the UNet (31), which allows for a pixel-wise transformation of an input image into an output image. Essentially, deep learning excels at computing an unknown transformation by using a large example dataset, often referred to as a training set. We hypothesize that UNet, or some other deep neural networks are able to upscale low resolution SI (LRSI), together with the T1-weighted (T1w) image, to produce high resolution SI (HRSI). To test this hypothesis the biggest challenge is that a large, publicly available SI dataset is unavailable and difficult to acquire experimentally. In order to create this data set, HRSI (128x128 pixels) and LRSI (16x16 pixels or some other low resolution) experiments would have to be performed on thousands of diverse patients with different pathologies, which is not feasible. Thus, it is seemingly impossible to perform deep learning for super-resolution SI.

In this paper, we report a novel work on the development of a deep learning technology capable of producing super-resolution spectroscopic images. An SI generator is used to produce LRSI and HRSI data in order to train and test a deep learning model. Using this data, a UNet taking advantage of densely connected layers (D-UNet) is built and trained. The inputs for the D-UNet are the T1w image and the low resolution SI image while the output is the high resolution SI. The results of the D-UNet reconstruction are compared both qualitatively and quantitatively to simulated and *in vivo* high resolution SI data.

## 2. Methods

### 2.1. Spectroscopic Imaging Dataset

Two different MRI data sets were utilized to produce synthetic SI data for developing the deep learning model. The first MRI data set comprised of 27 axial slices from the MATLAB MRI dataset. MR images as well as white matter (WM) and gray matter (GM) masks from the open access series of imaging studies (OASIS) project (32), which contained 416 axial images from subjects ranging in age from 18 to 96 years old, were also used. From these limited data, 102,169 SI datasets were synthesized using an SI generator, the details of which are found below.

### 2.2. Spectroscopic Imaging Generator

The SI generator was designed to address the lack of T1w images, as well as the lack of paired LRSI and HRSI data. First, the generator created augmented T1w (*aT*1*w*), white matter (WM), and gray matter (GM) images from an input T1w image. Then, the generator would produce a matched LRSI and HRSI for the *aT*1*w* image.

#### 2.2.1. Augmenting T1w Images

An input T1w image is first segmented into WM and GM images via an intensity based approach. First, the maximum WM intensity (WM_*max*_), and the minimum GM intensity (GM_*min*_) are determined from the image. Then, WM and GM images are made by applying the following:

(1) $$WM=\left(\frac{S-GM_{min}}{WM_{max}}\right)\;\cdot \;M$$

(2) GM=(1−WM) · M

Above, *S* is the original signal intensity of the input T1w image, and *M* is a mask for the brain region only, and is applied through an element-wise multiplication. The above equations ensure that the elements of both the WM and GM images range from zero to one, and are representative of the percentage of WM or GM present in any voxel.

Then, the SI generator modifies the input T1w image to produce an *aT*1*w* image. The contrast of the T1w image is altered by the following:

(3) $$aT1w=R\left(S_{n}^{r_{1}}+L\right)$$

Here, S_*n*_ is the normalized input T1w signal and r_1_ is a random number between 0.5 and 2.5. *R*() is a rotation and field of view (FOV) truncation transformation that rotates the image randomly in the range of –15° to 15° and randomly truncates the image in the range of 0 to 40 pixels in any direction. *L* is a matrix that represents up to 6 lesions of varying intensity, location, and size. Since this lesion matrix is random, the *aT*1*w* image may or may not contain any hyper-intense or hypo-intense regions. The same transformation used in Equation (3) is also applied to the WM and GM images.

#### 2.2.2. Production of Matched LRSI and HRSI Maps

In order to produce data useful for clinical applications, the SI generator operated under an assumption that is biologically valid: WM and GM regions of the brain have metabolism associated with biochemical concentrations (33). With this assumption, a given metabolite could be more concentrated in WM vs. GM, less concentrated in WM vs. GM, or equally concentrated in WM and GM regions.

Working with this biological assumption, a high resolution metabolite map is generated by adding a random ratio of the WM and GM images together:

(4) $$\begin{array}{r} HRSI=r_{2}*WM+\left(1-r_{2}\right)*GM+B+r_{3}*L \end{array}$$

In Equation (4), r_2_ is a random number between 0 and 1. *B* is a matrix that adds a random signal bias into the metabolite map, which helps to simulate the presence of more metabolite signal from the anterior or posterior, as well as the left or right brain regions. *L* is the same lesion matrix used in Equation (3), and *r*_3_ is a random number between –1 and 1.

Finally, the HRSI is downsampled to the desired low resolution via k-space truncation. Random noise is also added to this low resolution k-space data before a Fourier transformation is used to bring this data back to the spatial domain. Next, the low resolution image is upscaled to the same resolution as the HRSI using nearest-neighbor interpolation to yield the final low resolution SI.

It is important to note that because of the variables *r*_1_, *r*_2_, *r*_3_, and *L*, it is possible to produce several different matched *aT*1*w* images, HRSI, and LRSI from the same input T1w image. In addition, the same *aT*1*w* image can give rise to a large number of matched HRSI and LRSI, and thus this transformation is a one to many transformation. Therefore, a single input T1w image can produce hundreds of unique datasets for training a deep learning model.

### 2.3. Densely Connected UNet (D-UNet) Architecture and Training

**Figure 1:**
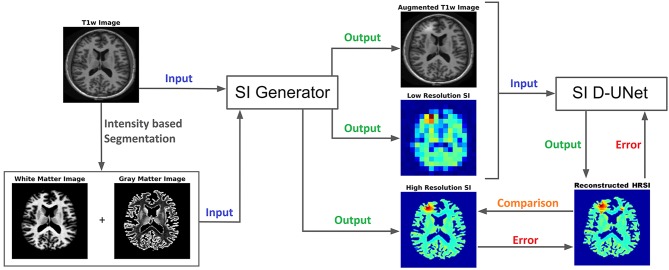
The workflow for training the D-UNet model is shown. The SI generator provides a dataset consisting of an augmented T1w image, a low resolution spectroscopic image, and a ground truth high resolution spectroscopic image. The spectroscopic images already show the distribution of a particular metabolite (or the distribution of a particular spectral point), such as choline, and therefore do not contain a spectral dimension. Then, the network transforms the *aT*1*w* (128x128 pixels) and LRSI (128x128 pixels after nearest-neighbor interpolation) into an initial HRSI reconstruction (128x128 pixels). In the example above, the LRSI and HRSI reconstruction have in-plane spatial resolutions of 1.4 × 1.4 cm^2^ and 1.7 × 1.7 mm^2^, respectively. This reconstruction is compared to the ground truth, and the mean squared error is calculated. Utilizing this error, the model changes the weighting parameters for the features, and continues training by using a different dataset. After training on 102,000 datasets, the model weights are refined and the reconstruction errors are minimized.

**Figure 2:**
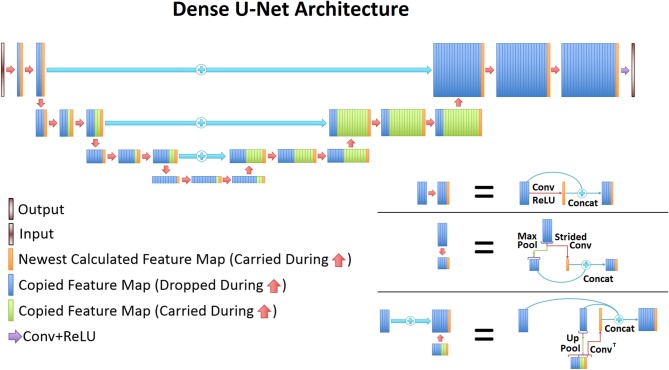
The general D-UNet architecture is displayed. Each forward convolution consisted of a convolutional layer and a concatenation process. This concatenation carries over important features which can be used to make the next layer more intelligent. In addition to local concatenations, certain features were concatenated to deeper layers in the network. More specifically, every feature map that is produced from a convolution is carried over to the end. Maxpooled features are not, since a higher resolution of the feature already exists. This allows for prior information to improve the overall reconstruction quality. In order to use the most information possible, the last convolutional layer contains all of the carried over features.

The UNet architecture (31) is typically implemented for segmentation purposes, however it primarily operates by performing pixel-wise transformations on input images, which is applicable to the SI super-resolution problem. Using standard convolutional and max pooling layers, the UNet first continuously convolves and pools the input image until the image reaches a small size, which aids in extracting valuable global features. Next the image is scaled up through a combination of up-pooling, transpose convolutions, and feature concatenations. This second process helps to identify vital local features so that the UNet can refine the image at a finer resolution. However, due to the number of features necessary for this process, the classical UNet suffers from extremely long training times, overfitting issues, and potential inefficiencies when tuning the weights. Therefore, this study utilized densely connected convolutional layers (34) to develop the novel densely connected UNet (D-UNet) architecture, and the workflow for training is shown in Figure 1. Densely connected networks carry over features from layer to layer, allowing for all previous information to be used for determining important features. The general architecture of the D-UNet used in this study is shown in Figure 2. The D-UNet utilized 32 feature maps at every max pooling layer. In addition, all convolutional layers made use of the ReLU activation function (30) and used a dropout (35) of 0.1. Certain features, shown in green and orange in Figure 2, were copied over to the following layers, and were also concatenated later on in the network. In total, three max pooling layers were used for the D-UNet. Since low resolution SI experiments can have diverse resolutions, three identical D-UNets were made to upscale low resolution spectroscopic images for acquisitions with 16x16, 24x24, and 32x32 spatial points.

The D-UNet required two inputs: a rescaled (128x128 points) T1w image and the corresponding LRSI image (16x16, 24x24, or 32x32 points) upscaled using nearest-neighbor interpolation (128x128 points). The predicted output of the D-UNet was a denoised HRSI image (128x128 points). For training, *aT*1*w*, HRSI, and LRSI were created from the SI generator, as described above. The Adam optimizer (36) was used with a learning rate set to 1 × 10^−3^, and mean squared error (MSE) was used as the cost function, which determined the difference between the D-UNet output and the desired output:

(5) $$\begin{array}{r} MSE=\sum \sum \frac{{\left(O-HRSI\right)}^{2}}{m^{2}} \end{array}$$

Above, *O* is the output of the D-UNet, *HRSI* is the true simulated high resolution SI, and *m* is the output dimension of the network, which in this case is 128. The summations are performed over both dimensions to yield a single value. The network was trained on an 8GB Quadro K5200 graphical processing unit (GPU) using the Keras (37) and Tensorflow (38) packages in Python 3.6.

Two datasets were made for the development and evaluation of the three D-UNets: a training dataset and a testing dataset. The training dataset comprised of 102,000 data from the SI generator using 135 axial images. The testing dataset used 169 different axial images (independent from the training set) from the OASIS project, and 169 matched *aT*1*w*, HRSI, and LRSI images were produced via the SI generator. Each of the three D-UNets were trained for a total of 102 epochs. For this study, an epoch was defined as a new set of 1,000 matched HRSI and LRSI data. The first two epochs were trained using a batch size of one to ensure that the network would not fall into a local minimum. The remaining 100 epochs were trained with a batch size of 10. Varying batch size in this manner has been shown to help reduce the number of epochs necessary for training, while also reducing the need for hyper-parameter tuning (39).

### 2.4. D-UNet Evaluation and Comparison Metrics

#### 2.4.1. Testing Set Evaluation

**Figure 3:**
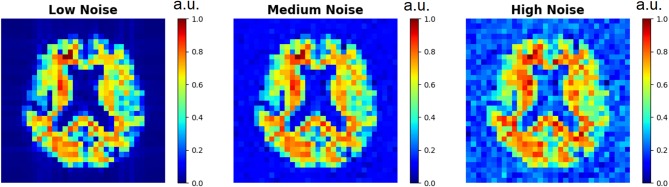
Low resolution spectroscopic images generated using the SI generator are shown. To show the effect of different random noise levels, all other random parameters were the same between the three images. Low noise level, medium noise level, and high noise level were classified as 2–5, 15–20, and 30–40% of the maximum signal intensity, respectively.

The three D-UNets evaluated all 169 matched images (*aT*1*w* and LRSI) to produce reconstructed high resolution spectroscopic images (*Recon*_16*x*16_, *Recon*_24*x*24_, and *Recon*_32*x*32_). These reconstructed images were compared to the ground truth HRSI using mean squared error. This process was repeated with varying noise levels inserted into the input LRSI in order to determine the role of noise on the reconstruction process. Example low resolution spectroscopic images can be seen in Figure 3. The reconstructed images were also compared to zero-filling and bicubic interpolation to assess the improvement of the D-UNet results over standard methods. For this comparison, both zero-filling and bicubic interpolation were applied to an LRSI of 32x32 points to generate the 128x128 interpolated images.

#### 2.4.2. Spectral Reconstruction Evaluation

In addition, the three D-UNets were used to reconstruct magnitude spectra point-by-point from low spatial resolution to high spatial resolution. Magnitude spectra were used because the model was not trained for evaluating real and imaginary numbers simultaneously. From the test set, a single subject was used to generate high resolution chemical maps of the major metabolites, including NAA, Glu, Gln, Cr, Ch, and mI. GAMMA simulation (40) was used to simulate the spectra for these metabolites using an echo time (TE) = 30 ms, spectral bandwidth of 2,000 Hz, and time points = 512 for a magnetic field strength (*B*_0_) of 3T. Also, the spectra were exponentially line broadened to roughly 8 Hz. These spectra were then distributed spatially based on their respective high resolution maps, and were transformed to produce LRSI. The T1w image and LRSI were input into the three D-UNets to produce *Recon*_16*x*16_, *Recon*_24*x*24_, and *Recon*_32*x*32_ spectral data. Two example spectra were extracted from these reconstructed images and compared to the simulated ground truth using mean squared error.

#### 2.4.3. *In vivo* Evaluation

Finally, high resolution spectroscopic images were acquired on a 7T whole-body MR scanner (Magnetom, Siemens Healthcare, Erlangen, Germany) using a previously published protocol (20). The Institutional Review Board (IRB) at the Medical University of Vienna approved the study and ten healthy volunteers (mean age = 31.7 years old) signed written and informed consent forms. All experiments were performed in accordance with relevant guidelines and regulations. The protocol utilized free induction decay based MR spectroscopic imaging (41) with TR = 200 ms for a total scan time of 21 min. After acquisition, residual lipids were removed using *ℓ*_2_ regularization (42) and the spectra were quantified using the LCModel (43) package to yield concentrations for several metabolites. Therefore, high resolution (128x128 pixels, 1.7 × 1.7 mm^2^) metabolite maps for NAA, Cr, Ch, Glu, Gln, and mI were obtained. These metabolite maps were down-sampled to 32x32 resolution images and were input into the 32x32 D-UNet along with corresponding T1w images to yield *Recon*_32*x*32_ for all datasets. These reconstructed images were then compared to the experimentally acquired HRSI using mean squared error as described in Equation (5). In addition, Glu/Cr and Ch/Cr ratios for both the reconstructed and experimentally acquired images were measured over all ten subjects. These ratios were investigated as a function of T1w intensity, which directly corresponds to the ratio of WM and GM in the brain. Finally, correlations between the reconstructed and experimental results were performed to yield the correlation coefficients (*r*) for the Glu/Cr and Ch/Cr ratios.

## 3. Results

### 3.1. Training Results

**Figure 4:**
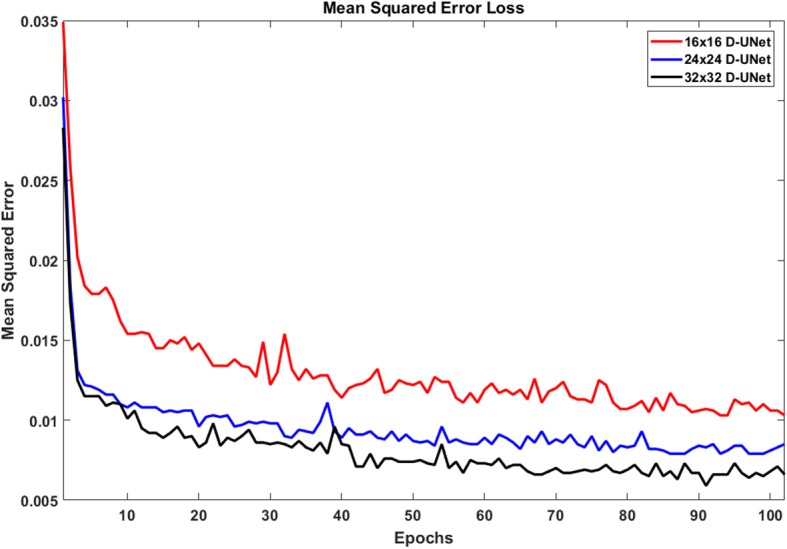
The loss functions for the 16x16 D-UNet (red), 24x24 D-UNet (blue), and the 32x32 D-UNet (black) are shown. All three loss functions drop significantly in the first 2 epochs, and then gradually decrease as the training continues. Overfitting is not an issue with the current training method, because each epoch contains a new set of 1,000 data. Therefore, the network does not see any dataset more than once. While more epochs could be used, the loss function flattens after 70 epochs, which implies that further training will yield minimal improvement.

Due to the novel D-UNet architecture, the mean squared error loss rapidly converged close to a reasonable value after only 2 epochs for all three networks, and the loss functions are shown in Figure 4. The loss continued to decrease with more epochs when a larger batch size was used for the remaining 100 epochs. From Figure 4, it is clear that the final loss was better for the 32x32 D-UNet than the 24x24 or 16x16 D-UNets. This is theoretically expected because higher initial resolution should aid in the estimation of unknown points, and this is true for conventional resolution enhancement techniques as well. While a low dropout was used in the architecture, overfitting was not a primary concern for the D-UNet training framework because of the reduced number of weighting parameters in the model. The results from the testing dataset also highlight the fact that the D-UNet training was generalized and applicable to never before seen data.

### 3.2. Test Set Results

**Figure 5:**
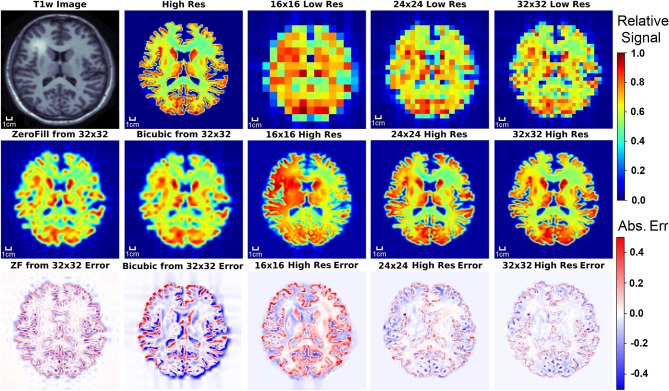
The results of the three D-UNets are shown for an example test subject. The augmented T1w image is used in conjunction with the three low resolution images as inputs for the three D-UNets. The reconstructed HRSI (16x16 High Res, 24x24 High Res, and 32x32 High Res) are shown below their respective low resolution images. In addition, zero-filling and bicubic interpolation were applied to the 32x32 LRSI to produce 128x128 interpolated images. Error maps are produced by subtracting the reconstructed images and the ground truth high resolution image (High Res). The 16x16 High Res displays much more error than the 24x24 and 32x32 High Res images. This is mostly due to better local signal refinement at the location of the lesion for the 24x24 and 32x32 reconstructions.

Figure 5 displays the results from the three different D-UNet reconstructions, as well as the results of the standard zero-filling and bicubic interpolation methods. In order to provide a more stringent comparison, both zero-filling and bicubic interpolation were applied to the 32x32 low resolution metabolite maps instead of the lower resolution 16x16 or 24x24 metabolite maps. All of the D-UNet reconstructions are able to determine the abnormally high signal from the lesion shown in the T1w image. While zero-filling outperforms both bicubic interpolation and the 16x16 D-UNet, both the 24x24 and 32x32 D-UNets yield better results than zero-filling.

**Figure 6:**
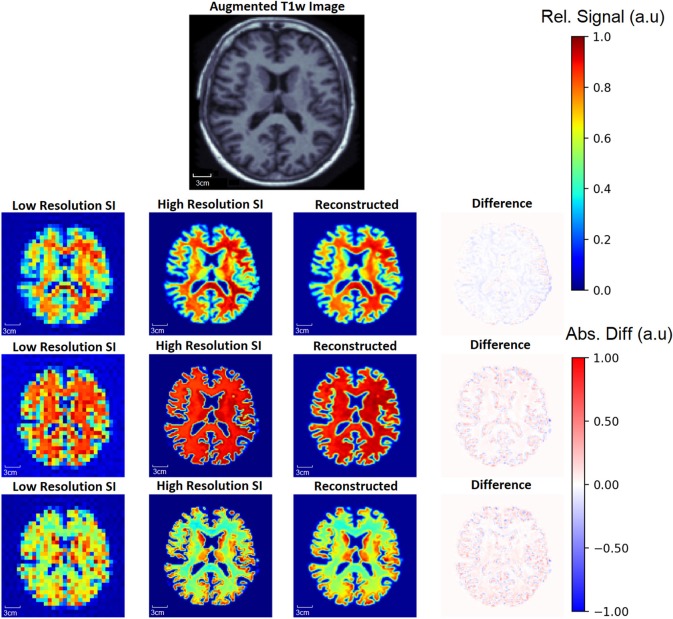
From one augmented T1w image, the generator is capable of producing multiple ground truth high resolution (High Resolution SI) images and low resolution (Low Resolution SI) images, a small sample of which are shown. In this example, the top row shows images where metabolite signal is higher in WM. In the middle row, the metabolite signal is equal in WM and GM, whereas the metabolite signal is higher in the GM in the bottom row. Since a single input T1w image can produce many augmented T1w images, the generator allows for an exponentially large number of unique training data. The reconstruction for each *aT*1*w* image and LRSI is performed with the 32x32 D-UNet to yield the reconstructed HRSI images (Reconstructed). The difference maps are produced by subtracting the reconstructed and ground truth images.

To demonstrate the capability of the SI generator, Figure 6 shows a sample of the possible images produced from the same *aT*1*w* image. The *Recon*_32*x*32_ images are also shown, as well as difference maps between the HRSI and *Recon*_32*x*32_. It is clear that the SI generator is capable of producing a wide variety of *SI* images that mimic biochemicals that are more prominent in GM, more prominent in WM, or equally prominent in both tissue types.

In addition, a quantitative comparison between these methods is shown in Table 1. Noise level was varied to determine the effect of noise on the super-resolution methods. Low noise level, medium noise level, and high noise level were classified as 2–5, 15–20, and 30–40% of the maximum signal intensity, respectively. From Table 1, the 32x32 D-UNet demonstrated the best performance at every noise level. At medium noise levels, the 24x24 D-UNet outperformed zero-filling, and at high noise levels both the 16x16 D-UNet and 24x24 D-UNet outperformed both zero-filling and bicubic interpolation.

**Table 1:** The mean squared error between the high resolution ground truth (HRSI) and several methods are tabulated.

**Method**	**Noise Level**		
	**Low**	**Medium**	**High**
Zero-Fill from 32x32	1.109	1.652	4.505
Bicubic from 32x32	2.794	3.129	3.820
16x16 D-UNet	1.863	2.420	2.761
24x24 D-UNet	1.139	1.316	1.745
32x32 D-UNet	**0.7460**	**0.9722**	**1.599**

*These values are the total sum of the mean squared error over 169 test subjects. The 32x32 D-UNet reconstruction outperforms all of the other methods. With higher random noise present in the LRSI, the 16x16 and 24x24 D-UNets outperform both zero-filling and bicubic interpolation. It is important to note that this is true even though the zero-filling and bicubic interpolation methods are applied to a 32x32 image. Bold values indicate the method with the lowest mean squared error for each comparison*.

### 3.3. Spectral Reconstruction Results

**Figure 7:**
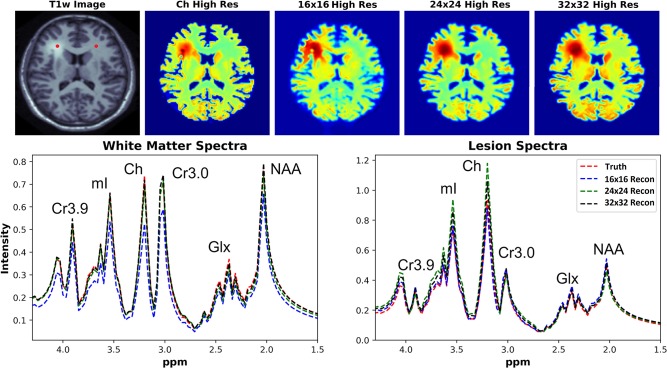
Magnitude spectral reconstructions using the three D-UNets are shown for two voxels. The voxel locations for the white matter and lesion spectra are displayed in the T1w image as red points. The spectra generated from the ground truth (red), the 16x16 (blue), 24x24 (green), and 32x32 D-UNets (black) for the 1.5–4.3 ppm range are displayed. For spatial comparison, the choline metabolite maps for each method are also shown. All metabolite maps are scaled from 0 to 1. The 24x24 and 32x32 D-UNet reconstructions over-estimate the amount of choline in the lesion. However, the 16x16 reconstruction under-estimates the amount of metabolite signal in the healthy white matter region.

The ability of the D-UNets to reconstruct spectra at high spatial resolutions are highlighted in Figure 7. The 32x32 D-UNet reconstructs the lesion and contra-lateral white matter spectra reliably. In contrast, the 16x16 D-UNet underestimates the white matter spectrum. The 24x24 D-UNet performs very similarly to the 32x32 D-UNet, however it overestimates the Ch and mI signals in the lesion spectrum by roughly 20%. Overall, the mean squared error for the healthy white matter spectrum was 0.34, 0.030, and 0.0085 for the 16x16 D-UNet, 24x24 D-UNet, and 32x32 D-UNet, respectively. For the lesion spectrum, the mean squared error was 0.051, 0.36, and 0.13 for the 16x16 D-UNet, 24x24 D-UNet, and 32x32 D-UNet, respectively. From a quantitative standpoint, all three D-UNets would be able to determine the abnormally elevated Ch, as demonstrated from the metabolite maps.

### 3.4. *In vivo* Results

**Figure 8:**
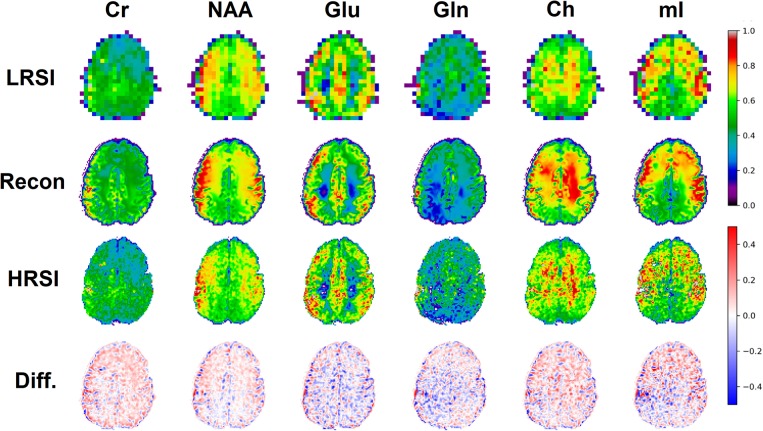
An *in vivo* example of a healthy volunteer is used to demonstrate the potential application for the D-UNet. The experimental high resolution SI (HRSI) data was acquired at 128x128 resolution using an accelerated acquisition protocol (20). This data was then down-sampled to produce 32x32 low resolution SI (LRSI) metabolite maps for Cr, NAA, Glu, Gln, Ch, and mI. Together with the T1w image, the low resolution metabolite images were used to reconstruct high resolution spectroscopic images (Recon) using the 32x32 D-UNet model. The difference maps between the Recon and HRSI images (Diff) are also shown.

**Figure 9:**
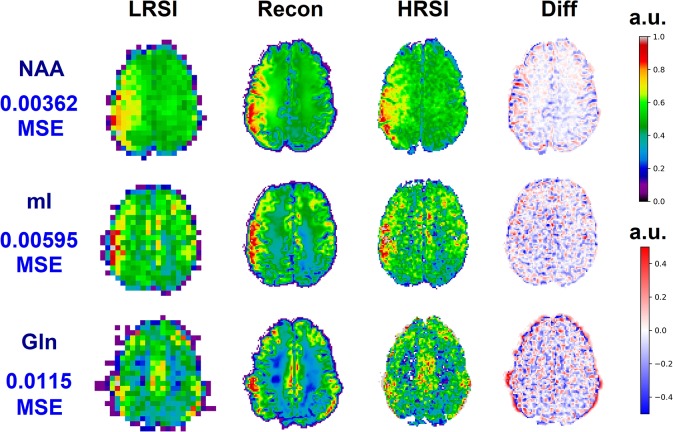
Three reconstructions showing low MSE (top), average MSE (middle), and large MSE (bottom) are shown for three different volunteers. In general, lower MSE were observed for metabolites with higher signal-to-noise ratios such as NAA, whereas larger MSE values were calculated for low SNR metabolites such as Gln. The different MSE values are highlighted by the errors seen in the difference maps.

**Figure 10:**
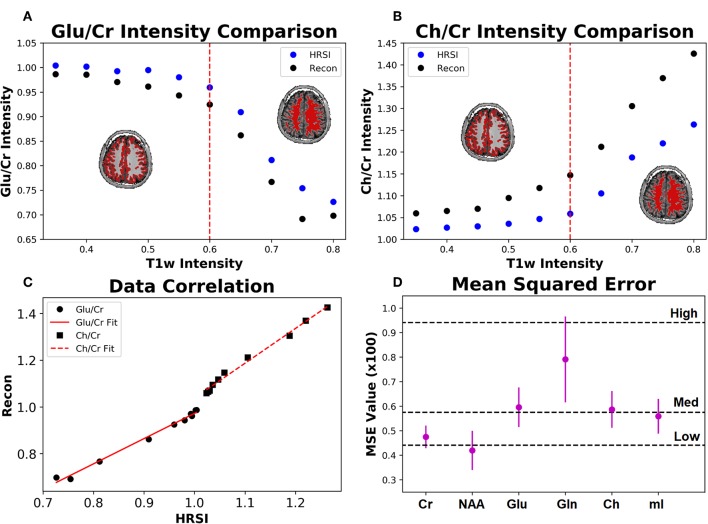
The Glu/Cr **(A)** and Ch/Cr **(B)** signals averaged over ten subjects are shown for the experimentally acquired images (HRSI) and the images reconstructed from low resolution 32x32 images (Recon). The signals are shown as a function of the T1w intensity, which is representative of the gray and white matter content of the voxel. The red dotted line represents the point at which the gray matter content equals the white matter content in a voxel. The correlation between the HRSI and Recon values are plotted **(C)** with linear fits. For both Glu/Cr and Ch/Cr, the r^2^ values of the fits are above 0.99. Finally, the mean squared error for the ten subjects calculated between the HRSI and Recon for each metabolite map **(D)** is displayed. The dotted black lines reflect the MSE from the testing set for different noise values (low, medium, and high).

The ability of the 32x32 D-UNet to reconstruct the LRSI of Cr, NAA, Glu, Gln, Ch, and mI for the *in vivo* data is shown in Figure 8. This figure shows the reconstructed images, experimental HRSI, and difference maps between the two for each metabolite for one healthy volunteer. All reconstructed images retain the metabolite signals from the low resolution maps, and also show regional changes similar to the HRSI. For example, Glu is more concentrated in the GM and less concentrated in the WM, which is a well-known regional difference in the brain (33). Another well-known regional difference is that Ch is more concentrated in WM regions, which is apparent in both the reconstructed and experimental images. Figure 9 shows reconstructions with low, average, and large MSE values. In general, lower SNR metabolites appeared to have a larger MSE value compared to higher SNR metabolites. From a quantitative standpoint, the average MSE values over the ten volunteers for Cr, NAA, Glu, Gln, Ch, and mI were 0.0048, 0.0042, 0.0060, 0.0079, 0.0059, and 0.0056 respectively. These errors are displayed in Figure 10D and plotted against the average MSE values obtained for the testing set using different noise levels (low, medium, high). It is clear that the MSE values are in most cases comparable to simulated test images with 2–20% noise, with the exception of Gln which is most comparable to test images with 35% noise.

Figure 10 also shows the Glu/Cr and Ch/Cr ratios as a function of the T1w intensity averaged over the ten volunteers. The ratios are taken after normalization of the metabolites as part of the super-resolution reconstruction, which is why Ch/Cr appears larger than Glu/Cr in the figure. The trend shows that with higher WM content, Glu/Cr decreases while Ch/Cr increases. The correlation between the experimental HRSI and Recon results are shown in Figure 10C. Quantitatively, both Glu/Cr and Ch/Cr ratios have high squared correlation coefficients, *r*^2^ > 0.99. This highlights the fact that important biological relationships are preserved in the reconstructed images.

## 4. Discussion

Although SI provides invaluable information regarding the biomolecular processes of tissues *in vivo*, experimental limitations have greatly hindered the integration of this method into standard clinical protocols. This study demonstrates a technique capable of overcoming one of the greatest challenges in SI, which is poor spatial resolution. By utilizing a deep learning framework, it is shown in Figures 5–9 that high resolution spectroscopic images can be produced from the combination of low resolution spectroscopic images and T1w images. In addition, as seen in Figure 7, it is possible to reconstruct spectra at higher spatial resolutions. The reconstruction method also preserves important regional metabolic differences and shows low errors for *in vivo* reconstructions, as shown in Figure 10. This deep learning super-resolution method was compared to both zero-filling and bicubic interpolation, and proved to be better than these methods for all noise levels.

Deep learning requires large datasets, which are not readily available for SI. Unfortunately, there is also a lack of ground truth for high resolution spectroscopic imaging due to the fact that experimental results may contain chemical shift displacement artifacts, B_0_ inhomogeneity issues, partial volume effects, low signal to noise ratios, water contamination, or other forms of signal contamination. It is also prohibitively long to scan at high resolution (128x128) without using several acceleration methods, making a ground truth impossible to obtain from the human brain with current technology. Therefore, an SI generator was developed to simulate training and testing data from a publicly available dataset. By including various probabilistic transformations, such as contrast variations, metabolic signal changes, and FOV variations, the SI generator was capable of providing a diverse and large dataset for the training of the three D-UNets. These data may not be entirely realistic, and this generator must be validated more rigorously in the future. For this study, the dataset does seem to be representative of real acquisitions, as seen from the *in vivo* results.

The *Recon*_32*x*32_ and HRSI experimental images are very similar, as seen from Figures 8–10. The reconstructed images show better resemblance to the anatomical T1w images, including cerebral spinal fluid localization. However, both the *Recon*_32*x*32_ and HRSI experimental images provide similar quantitative results, as seen in Figure 10. Theoretically, the *Recon*_32*x*32_ images would require $\frac{1}{16}th$ to $\frac{1}{4}th$ the time to acquire, depending on the acceleration methods implemented. Therefore, it is important to note that aside from super-resolution, the D-UNet may also be used as a means to accelerate a spectroscopic imaging protocol in the future. Additionally, the reconstructed *in vivo* images are denoised while retaining essential metabolic information for different tissues of the brain, which may be desirable for certain applications. While the simulated and *in vivo* data demonstrate that the reconstruction method is accurate, one of the main disadvantages of this work is that it has not been validated *in vitro*. This is due to the fact that a high resolution SI phantom similar to the human brain is not available. Since the D-UNet model is trained using *in vivo* anatomy, it is not capable of reconstructing high resolution images from unrealistic geometries. Therefore, future work will focus on the development of a realistic, high resolution SI phantom for validation.

Even though the D-UNets outperformed zero-filling and bicubic interpolation, these models may not be perfect for HRSI reconstruction primarily due to experimental imperfections. As seen from Table 1, error increases as a function of noise. Intuitively, chemicals that are found in the body at lower concentration may have larger reconstruction errors than chemicals with higher SNR, which is also supported by the *in vivo* results shown in Figure 9 where the Gln reconstructed images have higher error than the other metabolite images. Therefore, prediction accuracy is limited by the quality of the original LRSI. Also, while the *in vivo* results have low mean-squared errors, it is important to note that down-sampling from a high resolution acquisition decreases potential acquisition problems such as lipid contamination and partial volume effects. Therefore, it is expected that a prospectively acquired low resolution data set will yield higher errors when reconstructed using the D-UNet. This must be evaluated in a more rigorous study where both low resolution and high resolution experimental SI data are acquired.

Of course, the original resolution of the experimental SI plays a large role in the reconstruction process. While 24x24 and 32x32 matrices provide relatively accurate high resolution reconstructions, the 16x16 resolution does not perform as well. This suggests that there is a lower bound necessary to accurately upscale high resolution SI. This might be true for other super-resolution techniques (21), so a more thorough comparison between this deep learning method and other methods may aid in identifying this lower bound. Furthermore, results may be biased by the quantitative methods implemented to produce the LRSI before the super-resolution process is performed. This bias could be removed in the future by developing a deep learning based approach to metabolite quantitation (44). However, it may be worthwhile to explore the differences between common one dimensional spectral quantitation programs, such as LCModel (43) or TARQUIN (45), on the upscaling process.

From the spectral reconstruction results shown in Figure 7, it is apparent that some metabolites are over- and under- estimated during the reconstruction process. Therefore, clinical diagnosis based on the D-UNet reconstruction must be made with caution, as results from this method could lead to false positives or false negatives. Before basing diagnosis on the D-UNet reconstruction, the process should be evaluated *in vivo* in a well-known brain cancer pathology to assess the rates of false positives or false negatives detected by experienced radiologists in the field.

The deep learning method presented in this study may be useful for other super-resolution transformations in the field of medical imaging. This is especially true for spectroscopic imaging of other nuclei, such as ^13^C and ^31^*P*, where lower SNR results in low spatial resolution acquisitions. Recently, accelerated hyper-polarized ^13^C spectroscopic imaging has shown to be promising for imaging prostate cancer (46, 47), and this technique could benefit by using the D-UNet model. In addition, ^31^*P* spectroscopic imaging has also been used to image cancer (48, 49). The main drawback, again, is the lack of SNR to adequately acquire high spatial resolution data. High resolution acquisition schemes have been proposed for ^31^*P* spectroscopic imaging (50), and the D-UNet model could provide an alternative for improving spatial resolution. The same SI generation process could be used for training for these other nuclei, however different anatomical sites must be included (breast, prostate, etc.) to yield accurate results depending on the desired application.

The same principles discussed in this work may also apply to positron emission tomography (PET) (51). It is well-known that the radioactive tracer is more prominent in certain tissues and lesions, and positrons from this tracer travel some distance before annihilating to produce the PET signal. The distance between the source and the annihilation can be thought of as a partial volume effect. This model can potentially be used to learn how to remove this partial volume effect artifact, and this would be applicable for CT-PET or MR-PET acquisitions. Ultimately, this deep learning model allows for the acquisition of high quality images without increasing the scan time or improving the hardware of the imaging system.

## 5. Conclusion

The D-UNet model presented in this study allows for the reconstruction of accurate super-resolution magnetic resonance spectroscopic images from the human brain. Utilizing this method, we demonstrate that a simulated, low resolution chemical map can be transformed together with the T1w image to produce a high resolution chemical map. This method demonstrates better accuracy than typical zero-filling and bicubic interpolation methods. Furthermore, we demonstrate that the accuracy of this model holds when evaluating our method on retrospective *in vivo* data. This model still needs to be validated on prospective *in vivo* data in the future. After further *in vitro* and *in vivo* validation, this method may be utilized for denoising, scan acceleration, and improved tissue delineation.

## Data Availability Statement

The datasets generated for this study are available on request to the corresponding author.

## Ethics Statement

The Institutional Review Board (IRB) at the Medical University of Vienna approved the study and ten healthy, adult volunteers signed written and informed consent forms prior to imaging studies. All experiments were performed in accordance with relevant guidelines and regulations of the IRB.

## Author Contributions

ZI and SJ conceived the experiments. DN designed the deep learning architecture. ZI and DN conducted the deep learning experiments. GH, SM, and WB acquired and processed the *in vivo* data. ZI and SJ analyzed the results. All authors reviewed the manuscript.

### Conflict of Interest

The authors declare that the research was conducted in the absence of any commercial or financial relationships that could be construed as a potential conflict of interest.
